# Influence of Inner Lining Atoms of Multilayered Hexagonal Boron Nitride Porous Membrane on Desalination

**DOI:** 10.3390/mi16050530

**Published:** 2025-04-29

**Authors:** Chulwoo Park, Daejoong Kim

**Affiliations:** Department of Mechanical Engineering, Sogang University, Seoul 04107, Republic of Korea; chulwoo87@sogang.ac.kr

**Keywords:** graphene, porous nanosheet, multilayered membrane, water transport, boron nitride, hexagonal boron nitride

## Abstract

Recent findings have demonstrated that the desalination and purification of contaminated water and the separation of ions and gases, besides solutions to other related issues, may all be achieved with the use of membranes based on artificial nanoporous materials. Before the expensive stages of production and experimental testing, the optimum size and form of membrane nanopores could be determined using computer-aided modeling. The notion that rectangular nanopores created in a multilayered hexagonal boron nitride (h-BN) membrane in a way that results in different inner lining atoms would exhibit unique properties in terms of the water penetration rate is put forth and examined in the current study. Nanopores in boron nitride sheets can be generated with the inner lining of boron atoms (B-edged), nitrogen atoms (N-edged), or both boron and nitrogen atoms (BN-edged). In this study, we compared the three different inner-lined nanopores of boron nitride nanosheets to a comparable-sized graphene nanopore and evaluated the water conduction.

## 1. Introduction

Due to the rapid increase in the world’s population, industrialization picking up speed, and environmental pollution, the recent clean water issue has garnered a lot of attention around the world [1,2,3,4]. One of the most significant issues of our time is finding adequate and clean water supplies [5]. The desalination of saltwater and decontamination of contaminated water are two processes that have recently attracted a lot of attention. There are several different techniques for decontaminating water, including flocculation, adsorption, chemical oxidation, and photocatalysis [6,7,8,9,10,11,12,13,14]. For desalination, there are three basic categories of water purification technologies, namely membrane technologies, distillation processes (thermal technologies), and chemical approaches [15]. Membrane technologies use either pressure-driven or electrical-driven types. Technologies utilizing pressure-driven membranes include reverse osmosis (RO), nanofiltration (NF), ultrafiltration (UF), and microfiltration (MF) [16,17,18,19]. The RO method has the benefits of no phase transitions, minimal energy usage, and high desalination efficiency among pressure-driven membrane methods [20]. The commercial RO method membrane design commonly uses polyamide membranes and cellulose acetate membranes. Polyamide membrane composites that are used in the commercial RO method were developed thirty years ago. The water flux of the material has only increased about two-fold in the past 20 years, and the performance of commercial RO membranes has not improved significantly in terms of selectivity and permeability [21,22,23,24]. Existing synthetic membranes suffer a ubiquitous, pernicious trade-off: highly permeable membranes lack selectivity, and highly selective membranes lack permeability [23].

However, materials with both high permeability and high selectivity are beginning to emerge. To increase the water flux of RO membranes, a wide range of nanomaterials have been tested. Nanomaterials such as graphene, carbon nanotubes (CNTs), boron nitride nanotubes (BNNTs), and boron nitride sheets are the commonly studied nanomaterials for desalination as they show improved water flux.

Researchers have discovered that single-layer porous graphene membranes produce significant levels of water flux [25,26,27,28]. But making single-layer graphene membranes was difficult, and the cost of creating large-area single-layer graphene membranes is still very high. Also, the single-layer material is prone to cracks and overlapping of graphene sheets, which prevents the practical application of single-layer porous graphene membranes in RO seawater desalination. This is true even though many studies have looked into the continuously improving preparation methods of graphene, such as particle bombardment and chemical etching [29,30,31]. This holds true even when producing single-layer boron nitride nanosheets (BNNSs) for RO membranes. BNNSs’ mechanical characteristics are comparable to those of graphene sheets [32]. Compared to single-layer sheet membranes, multilayer membranes are easier to prepare, have higher efficiency, and are less expensive [24]. Research conducted in 2013 showed that multilayered graphene exhibits hydrophobicity larger than single-layered graphene. It also showed that multilayered graphene membranes can trap salt ions and exhibit better salt ion rejection when compared to single-layered membranes [33]. To study their desalination potential, many molecular dynamics (MD) investigations have been conducted on single-layered graphene, multilayered graphene, and single-layered boron nitride sheets; however, the number of studies on multilayered boron nitride is minimal.

A recent study conducted on ultrafiltration-grade polysulfone-based mixed matrix membranes (MMMs) incorporating two-dimensional boron nitride nanosheets (BNNSs) revealed that by adding BN to the membrane matrix, water permeability and humic acid rejection significantly increased as a result of an increase in pore size and surface negative charge [34]. The presence of grain boundaries and surface charge can cause a decrease in the water permeation performance of hBN membranes, according to recent research on water transport through nanopores in bi-crystalline hBN [35]. A 2018 study found that adding hydrophilic chemical functionalities like fluorine (-F) and hydroxyl (-OH) to nanoporous boron nitride nanosheet (BNNS) membranes can increase water permeability even at low pressures [36]. According to another study conducted in 2018, C-doped BN membranes can be favorably compared to the MoS2 membranes. C-doped BN’s high carbon content clusters water molecules at membrane pores, which lowers the hydration free energy and pore energy barriers and increases water flux through the pores [37].

The structural analogs of graphene are hexagonal BN sheets [38,39]. Multilayered boron nitride nanosheets have a graphite-like structure in a regularly stacked planar network of BN hexagons. An h-BN has an equal number of boron and nitrogen atoms [38]. It differs from graphene in that it is resistant to heat, is chemically inert, and does not conduct electricity. The fact that it appears white gives it the name “white graphite” as well [40,41,42,43,44,45]. In a study published in 2020, researchers discovered that the water transport processes of the armchair and zigzag hexagonal boron nitride (hBN) nanosheets with slit-like orifices are sensitive to edge patterns and are caused by fascinating electrostatic interactions between hBN and solution particles. The armchair nanoslits hardly distinguish between ions and water molecules, while the nanoslits with zigzag edges function together to prevent ion permeation and enable rapid water conduction [46]. In comparison to a pore with nitrogen atoms on the edge functionalized with hydrogen atoms, a boron nitride sheet with a nanopore with boron atoms on the edges functionalized with hydrogen atoms of the pore demonstrated improved desalination performance in terms of increased water flux [47]. In an investigation carried out to remove Hg^2+^ from industrial wastewater contaminated with Hg^2+^ using a triangle-shaped nanoporous boron nitride membrane, the researchers discovered that a N-edged pore encourages water molecules to pass through the nanopore when compared to a B-edged triangular pore [48].

As mentioned earlier, a previous work [46] was carried out on armchair and zigzag membranes with rectangular pores, pores that have both boron and nitrogen inner lining. The other work [48] that used a triangular pore shape investigated only boron inner-lined and nitrogen inner-lined nanopores. It is possible to create nanopores in boron nitride sheets with a boron edge (B-edged), a nitrogen edge (N-edged), or both a boron and nitrogen edge (BN-edged). So, in this work, we have investigated B-edged, N-edged, and BN-edged multilayered boron nitride nanosheet membranes with rectangular pores for water transport and have compared the results with those of a multilayered graphene membrane with a rectangular pore of similar size.

## 2. Simulation Model and Methods

**Figure 1:**
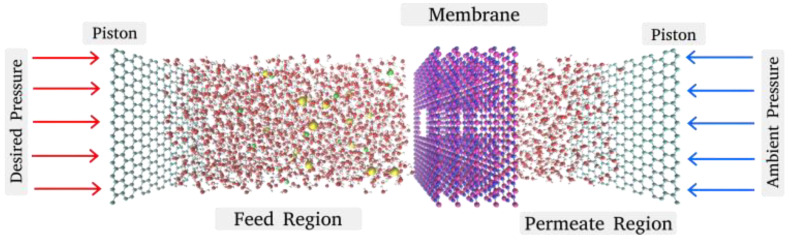
Simulation domain [magenta—boron atoms, blue—nitrogen atoms, cyan—carbon atoms, yellow—Cl^−^ ions, green—Na^+^ ions, red—oxygen atoms, white—hydrogen atoms].

In this study, SAMSON 2022 R1 (Software for Adaptive Modeling and Simulation of Nanosystems) software [49] was utilized to model the nanoporous multilayered membranes used in this investigation. The nanoporous multilayered membrane system used in this study consists of 5 layers. With a 3.5 Å interlayer space between the layers, these membrane layers are densely packed. On both sides of the nanoporous multilayered membrane structure, there are water molecules followed by the pistons. The graphene sheets that serve as the pistons on both ends have a size of ≈30 × 30 Å. In order to reduce the computational expense, the nanoporous membrane used in this simulation was kept frozen. Figure 1 depicts the simulation setup that was employed in this investigation.

The size of the overall simulation box used in this study has a dimension of 30 Å × 30 Å × 210 Å. When the system is modeled for the investigation that involves fresh water, a total of 2140 water molecules are used, of which 1712 water molecules are in the feed region and 428 water molecules in the permeate region. For the investigation that involves saline water, a total of 2052 water molecules are used, of which 1624 are in the feed region along with 16 Na^+^ ions and 16 Cl^−^ ions. In this study, the TIP3P water model was chosen from various available models. Park et al. have published a detailed discussion on the selection and application of different water models [50]. Table 1 shows the list of the force field parameters employed in this investigation.

To determine the Lennard–Jones (L-J) interactions between the various atoms employed in this work, we applied the Lorentz–Berthelot mixing rule. The simulations were run for 10 nanoseconds with a timestep of 1 femtosecond. To determine the long-range electrostatic interactions, we employed a pppm (particle–particle particle–mesh)-style solver [51]. The L-J interaction cutoff employed in this investigation is 10 Å. The simulations are performed using the LAMMPS software 2020 [52]. For the visualization of the simulation system, we used VMD 1.9.4a55 (visual molecular dynamics) software [53]. In this work, the canonical ensemble NVT and the Nosé–Hoover thermostat [54] are used along with SHAKE algorithm to constrain water molecules [55].

**Figure 2:**
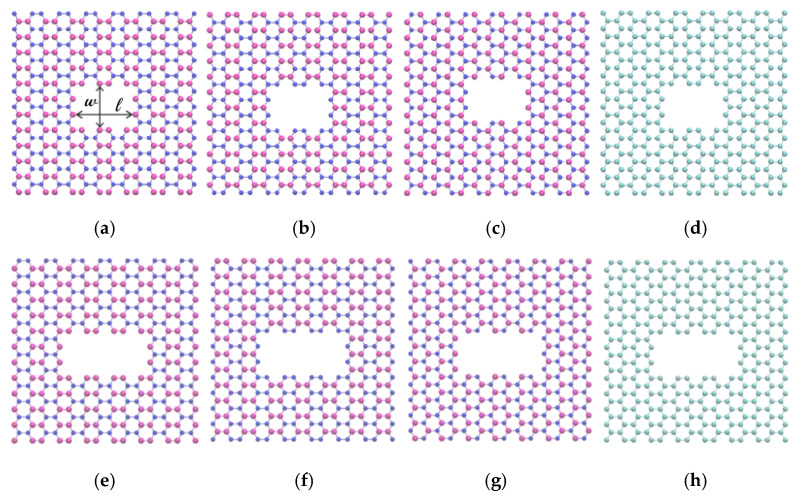
Schematics of the pores: (**a**–**d**) small pore, (**e**–**h**) large pore, (**a**,**e**) B-edged boron nitride, (**b**,**f**), N-edged boron nitride, (**c**,**g**) BN-edged boron nitride, (**d**,**h**) C-edged graphene [magenta—boron atoms, blue—nitrogen atoms, cyan—carbon atoms].

In this study, to simulate the pressure-driven flow, the piston at the end of the feed area is subjected to a pressure of approximately 150 MPa, while the piston at the end of the permeate region is maintained at atmospheric pressure [56]. Since the time scales for flow scale linearly with applied pressure, prior studies [27] have demonstrated that the results will be true at low pressures as well, despite a pressure of 150 MPa being substantially higher than that of an average desalination system, which is only a few MPa. In this study, two crucial characteristics for water transport were examined: the pore size and the inner lining atoms of the pore. We used two different pore sizes for our investigation, each with a B-edged boron nitride membrane, N-edged boron nitride membrane, BN-edged boron nitride membrane, and C-edged graphene membrane. The schematic of the pores used in this work is given in Figure 2. The larger pore is created by the removal of 30 atoms per layer of the membrane, and the smaller pore is created by the removal of 20 atoms per layer of the membrane. We also used both porous boron nitride nanosheets as well as porous graphene nanosheets for the membrane construction. As the bond length of the carbon atoms in the graphene sheet is different from the bond length between the boron and nitrogen atoms of the boron nitride nanosheets, the effective pore size also varies between the pore created in the graphene and the pore created in the boron nitride nanosheets. The dimensions of the rectangular nanopores used in this work are listed in Table 2.

## 3. Results and Discussion

### 3.1. Occupancy and Free Energy of Occupancy Fluctuations

**Figure 3:**
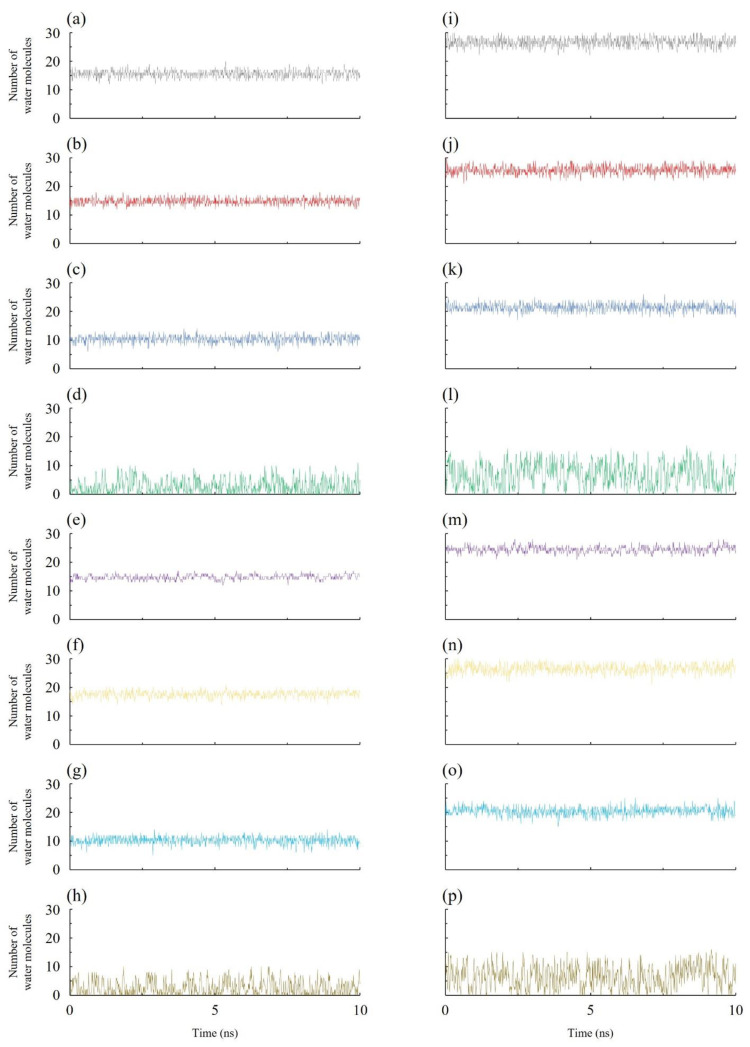
Number of water molecules inside different porous membranes: (**a**) B-edged small pore, (**b**) N-edged small pore, (**c**) BN-edged small pore, (**d**) C-edged small pore, (**e**) B-edged small pore with ions, (**f**) N-edged small pore with ions, (**g**) BN-edged small pore with ions, (**h**) C-edged small pore with ions, (**i**) B-edged large pore, (**j**) N-edged large pore, (**k**) BN-edged large pore, (**l**) C-edged large pore, (**m**) B-edged large pore with ions, (**n**) N-edged large pore with ions, (**o**) BN-edged large pore with ions, and (**p**) C-edged large pore with ions.

The occupancy of water molecules inside the nanopore aids in our understanding of the free energy within [57]. The shape and structure of the pore have a considerable impact on the water occupancy inside it. Figure 3 depicts how many water molecules are found inside each nanopore of the various membranes. It is evident that there are significantly more water molecules inside the nanoporous boron nitride nanosheet instances than their graphene counterparts. The fact that the effective pore volume of the graphene nanopore is smaller than that of the boron nitride nanopore is a major factor in this. When compared to the nanopores of boron nitride nanosheets, the effective pore area of graphene’s pores is roughly 3.7% less.

**Figure 4:**
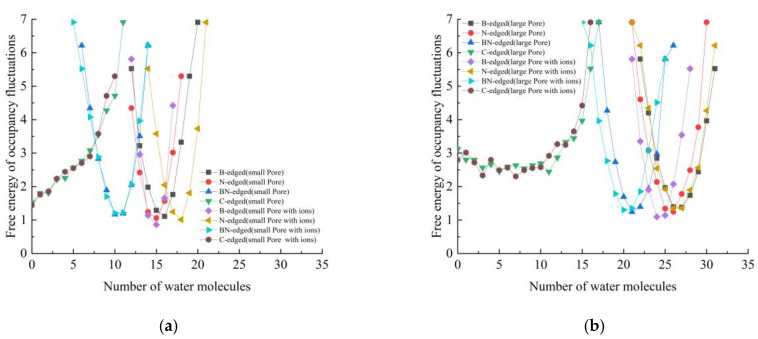
Free energy of occupancy fluctuations of water molecules inside different porous membranes: (**a**) small pore, (**b**) large pore.

The free energy of occupancy fluctuations of water molecules inside the nanopore of different membranes used in this study are given in Figure 4. Free energy of occupancy changes have been extensively discussed in numerous earlier works [50,57,58,59,60]. For the smaller pore configurations, the highest water molecule occupancy inside the nanopore is observed in the N-edged nanopore with its feed region having ions with 21 water molecules, while the graphene nanopore exhibited numerous empty states. All the BN configurations showed partially and fully filled states. No empty states were observed in these configurations. The most probable number of water molecules varied from case to case. The larger pore also exhibited similar kinds of occupancy states to those of the smaller pore configurations. The highest number of water molecules observed inside the nanopore is 31. Similar to the small pore configuration, only the graphene nanoporous membrane showed empty states. This shows that the flow of water molecules through the multilayered nanoporous graphene membrane occurs in a pulse-like manner.

### 3.2. Density Profiles

**Figure 5:**
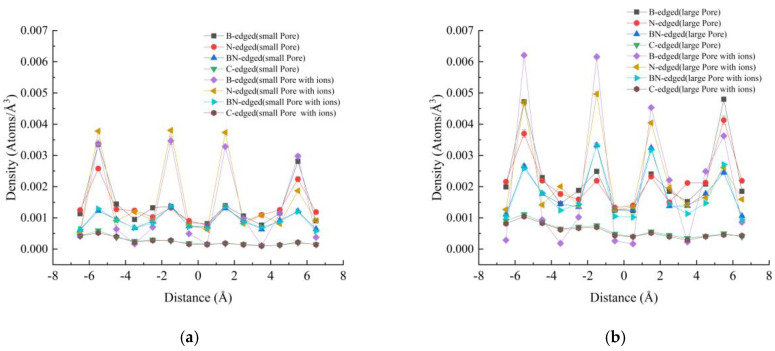
Density of oxygen atoms inside different porous membranes: (**a**) small pore, (**b**) large pore.

Density profiles of the oxygen atoms inside the nanopore for the different membranes and pore sizes used in this study are shown in Figure 5. From these density profiles, the difference depending on the particle’s charge can be observed. The graphene membrane, composed of electrically neutral particles, shows relatively flat profiles without any peaks, whereas the BN membrane, made of charged particles, exhibits significant peaks in oxygen density at various locations inside the nanopore. The calculations that include Na+ and Cl- ions show larger peaks compared to those without these ions, indicating that the ions trapped in the membrane lead to the stagnation of water molecules. These peaks result from the stagnation of water molecules inside the pore, caused by the ions trapped within the membrane pores. The trapped ions not only reduce the effective volume of the nanopore but also slow down the movement of water molecules due to the increase in the energy barrier inside the nanopore. Consequently, this results in the clogging of the nanopore. Even though the clogging observed inside the BN-edged nanopore is relatively low, the clogging observed inside the B-edged and N-edged nanopores is relatively high.

### 3.3. Water Conduction

**Figure 6:**
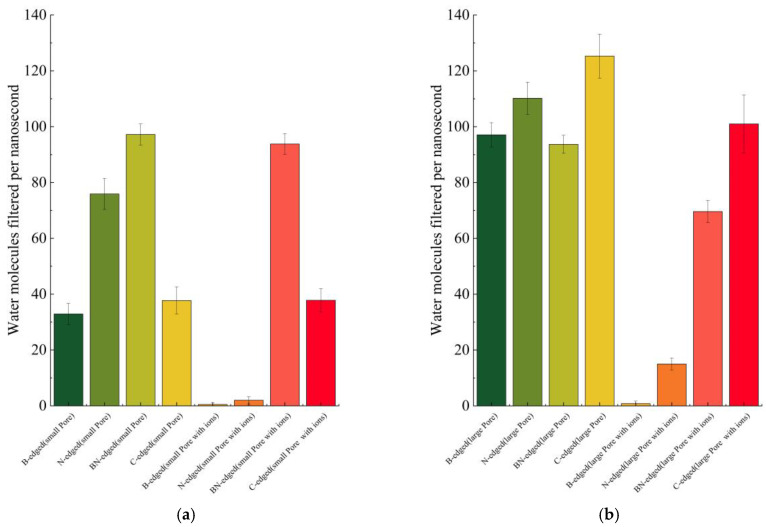
Water molecules filtered using different porous membranes: (**a**) small pore, (**b**) large pore.

The plot of the number of water molecules filtered by nanoporous boron nitride with different inner lining atoms of the graphene nanopore for two different pore sizes is given in Figure 6. From the plot, we can observe that the N-edged pore shows better water transport than the B-edged pore. This is similar to the observations from previous studies [48,61] as the nitrogen atoms of the porous boron nitride nanosheet and water molecules have a greater van der Waals (vdW) interaction than boron atoms. A 2007 study [61] reveals that, although vdW attractions between water molecules and boron atoms (ε_B-O_ = 0.5082 kJ/mol) are stronger than those between water molecules and carbon atoms (ε_C-O_ = 0.4340 kJ/mol), they are still not strong enough to allow water molecules to enter the BN pore. Water conduction in the BN pore is primarily driven by vdW interactions between water molecules and nitride atoms (ε_N-O_ = 0.6277 kJ/mol). Another key observation is the decrease in the amount of water filtered when the ions are introduced in the feed region of the boron nitride cases. This reduction is due to the ions that become trapped inside the membrane pore. In the B-edged pore, we found that numerous Cl^−^ ions were trapped inside the pore, whereas the Na^+^ ions become trapped easily inside the N-edged pore. These observations are similar to a previously reported work [62]. The occluded pore was the significant reason for reducing the transport of water molecules in the BN-edged large pore membrane. The armchair edges of the pore are another major factor contributing to the reduction in water transport through the membrane [63]. Previous studies discovered that nanoporous multilayered BN has lower water surface tension than nanoporous multilayered graphene [64]. The long-range wetting of the interfacial water that causes this decrease in surface tension also causes a reduction in water transport through the multilayered BN nanopore as compared to the multilayered graphene nanopore.

## 4. Conclusions

In this work, we performed non-equilibrium molecular dynamics simulations to understand the water transport and ion rejection of nanoporous multilayered boron nitride sheets with pores having different inner lining atoms. Our study shows that water transport through the membrane is contingent on the atomic type of the inner lining atoms of the boron nitride pores. As the BN nanosheets are formed with boron and nitrogen atoms, which have partial charges, the nanopores that are created have unique characteristics. Consequently, this also leads to undesirable qualities such as the trapping of ions inside the pore that results in clogging of the pore. By properly functionalizing the pore, undesired properties like the trapping of ions can be altered into desirable unique properties. By increasing the interlayer spacing of the nanosheets, it is conceivable to enhance the water transport phenomenon of the multilayered nanoporous boron nitride sheets.

## Figures and Tables

**Table 1:** Force field parameters.

	σ (Å)	ε (kcal/mol)
O	3.178	0.15587
B	3.453	0.0949
N	3.365	0.1448
C	3.3997	0.0859
Na	2.217	0.3519
Cl	4.849	0.01838

**Table 2:** Dimensions of the pores used in this study.

Type	Length (Å)	Width (Å)
B-edged (small pore)	10.12	7.51
N-edged (small pore)	10.12	7.51
BN-edged (small pore)	10.12	7.51
C-edged (small pore)	9.93	7.37
B-edged (large pore)	14.46	7.51
N-edged (large pore)	14.46	7.51
BN-edged (large pore)	14.46	7.51
C-edged (large pore)	14.18	7.37

## Data Availability

The original contributions presented in the study are included in the article, further inquiries can be directed to the corresponding author.
